# Amplified psychological reactions to war predict increased sleep problems among holocaust descendants

**DOI:** 10.3389/fpsyt.2026.1900539

**Published:** 2026-07-08

**Authors:** Ephraim S. Grossman, Amit Shrira, Lee Greenblatt-Kimron, Yuval Palgi

**Affiliations:** 1 Department of Education, Ariel University, Ariel, Israel; 2 Department of Social and Health Sciences, Bar-Ilan University, Ramat-Gan, Israel; 3 School of Social Work, University of Haifa, Haifa, Israel; 4 Department of Gerontology, University of Haifa, Haifa, Israel

**Keywords:** ancestral trauma, holocaust, intrusive linkage, PTSD, sleep, war

## Abstract

**Background:**

Ancestral trauma may result in increased sensitivity to adverse events among offspring. This increased sensitivity may be expressed in heightened PTSD symptoms, and by experiencing intrusive linkage between ancestral trauma and current traumatic events experienced by offspring. Sleep problems are another major consequence of traumatic events, but they have been underexplored in the context of intergenerational effects of trauma. Therefore, the current study examines the role of intrusive linkage and PTSD symptoms as potential mechanisms that may increase sleep problems following the October 7, 2023, attack and subsequent war among Holocaust descendants.

**Methods:**

The study included 359 Israeli participants (T1 age=45.44 ± 12.85), who completed four waves of sampling before (T1-T2) and after (T3-T4) the October 7 attack. Sleep problems (T2-T4), PTSD symptoms (T3-T4), and intrusive linkage between the Holocaust and the Israel-Hamas War (T3-T4) were measured using standardized questionnaires.

**Results:**

PTSD symptoms, intrusive linkage, and sleep problems decreased from T3 to T4. A reciprocal nature of effects among PTSD symptoms, intrusive linkage, and sleep problems emerged. Indirect effects were found between Holocaust background (having Holocaust survivor grand/parents) and T4 intrusive linkage via T3 PTSD symptoms. Indirect effects were also found between Holocaust background and T4 sleep problems via T3 PTSD symptoms and via T3 intrusive linkage.

**Conclusion:**

The findings suggest that intrusive linkage and PTSD symptoms can serve as mechanisms for amplifying sleep problems among offspring of traumatized grand/parents. Therefore, sleep disturbances might be a key outcome of increased susceptibility to negative events, which can be linked to intergenerational trauma effects.

## 1 Introduction

Long-term effects of exposure to a traumatic event may include post-traumatic stress disorder (PTSD). Studies have also examined how exposure to a previous trauma can intrude on the exposure to a current trauma in the same person. For example, following the 2023 October 7 attack by Hamas on Israel (1), it was shown that Israeli veterans of the 1973 Yom-Kippur war, who reported intrusive thoughts of the Yom-Kippur war trauma in the aftermath of the attack, displayed higher rates of psychopathology even when pre-attack symptoms were taken into consideration (2). The authors elaborate on the importance of an intrusive linkage between previous and current traumatic events with reference to the stress resolution perspective (3). Accordingly, intrusive linkage reflects the psychological impact of previous trauma exposure and its aftermath on subsequent trauma exposure, rather than solely addressing mere exposure to the current trauma. Following, people who experience similarities between past and current traumatic events may feel more threatened and consequently display increased post-traumatic responses. Hence, after experiencing a prior trauma, a second traumatic event perceived to be similar to the first traumatic event may elicit intrusive thoughts, in which thoughts or feelings of the first experienced trauma intrude into the second, current trauma. The extent of this linkage also predicts PTSD following the second trauma.

Another issue that may rely on similarity between traumatic events is reflected by the intergenerational effects of trauma. As opposed to the previous issue of linking two traumas in the same person, we now address a two-person model that focuses on ancestral trauma being passed down to the next generation. In this case, a previous parental or ancestral trauma may impact the outcome of trauma exposure in offspring (4, 5). For example, Shrira et al. (6) found that probable PTSD of one’s ancestors (parents/grandparents) increased vulnerability among Holocaust survivors’ offspring. In this study, Holocaust descendants and non-Holocaust descendants did not differ on probable PTSD before the 2023 October 7 attack on Israel. However, two months after the attack, Holocaust descendants were at a higher risk of having probable PTSD compared to non-Holocaust descendants.

A potential crossover between traumas and generations via intrusive linkage may thus be examined, especially in cases where traumas are similar, such as the massive 2023 October 7 attack, which was contextualized in Holocaust terminology (7). However, when the current trauma is less similar to ancestral trauma, intergenerational effects may be limited (8). A central gap in the literature is whether the intergenerational bridging of ancestral trauma with offspring trauma can indeed be accounted for by intrusive linkage of the trauma. Thus, the first aim of the current study is to extend previous results showing that intrusive linkage within a person also exists in a two-person model in an intergenerational context. The present study will examine the case of Holocaust ancestral trauma to predict current offspring PTSD symptoms in relation to the war that erupted on October 7, 2023, via intrusive linkage.

Beyond extending the notion of intrusive linkage to a two-person model of intergenerational transmission, the current study has two more goals concerning sleep problems. Sleep is a central factor that may be affected by trauma (9). Sleep has a crucial role in maintaining day-to-day functioning; hence, its disturbance can affect many functions (10), for example, psychomotor and cognitive performance, affect (11, 12), physical and mental health, and emotion regulation (13). Such effects may manifest over long periods (14). It is well documented that sleep has a bidirectional relation with different aspects of life, including mental health (15), mood (16), and PTSD (17). Thus, PTSD symptoms can result in impaired sleep, and poor sleep can exacerbate PTSD symptoms. However, it is not yet known if and how intrusive linkage of traumatic events may affect sleep. This potential association may even explain an aspect of the sleep-PTSD association. Some sleep researchers (18), suggest that the PTSD–insomnia association relates to the hyperarousal symptoms of PTSD, which may result from exposure to trauma, and in turn impact sleep (19). Other researchers (20) emphasize the role of intrusive thoughts in mediating the relationship between emotional dysregulation and insomnia. In the reverse direction, sleep problems may be linked with increased risk for specific trauma symptoms (i.e., complex PTSD) (21). In such cases, poorer sleep quality is linked to impaired cognitive and executive functions (22), resulting in a diminished ability to inhibit maladaptive intrusive thoughts and impaired emotional memory consolidation. Raging unprocessed memories of the trauma may facilitate a higher risk for PTSD (23).

The potential association of intrusive linkage with sleep quality has not been explored. This gap raises two questions about sleep in this context that we wish to address. First intrusive thoughts impair the ability to sleep (24), and poor sleep reduces prefrontal cortex ability to inhibit racing intrusive thoughts (25). Moreover, Brewin (23, 26), postulates that flashbacks are a specific type of episodic intrusive memory which are a normal response to a traumatic event. Flashbacks reflect a sensory, perceptual form of memory rather than a well-processed conceptual one. Following, there should be reciprocal effects between PTSD symptoms, intrusive linkage, and sleep problems. PTSD symptoms and intrusive linkage predict subsequent sleep problems and sleep problems, due to less pre-frontal inhibitory processes would predict more subsequent PTSD symptoms and intrusive linkage.

The second question arising from this gap is whether intrusive linkage serves as a mechanism (i.e., mediation) by which ancestral trauma may affect current sleep. This assumption builds on the same findings that drove the formulation of the previous hypotheses. First, ancestral trauma should produce elevated intrusive linkage with the current similar trauma, especially when the current trauma echoes the Holocaust trauma (8, 27). Second, intrusive thoughts and intrusive linkage should impair sleep (20). Thus, intrusive linkage is now hypothesized to be a mechanism through which ancestral trauma may impair subsequent sleep during current trauma exposure. Similarly, we predict that PTSD symptoms corresponding to the current war would also mediate between ancestral Holocaust trauma and current sleep. This is again due to two factors. First, ancestral trauma may increase current PTSD levels (6). Second, current PTSD comprises both arousal and intrusiveness, both of which increase sleep problems (18). Thus, current PTSD should also mediate between ancestral trauma exposure and current sleep quality.

### 1.1 Hypotheses

(a) Holocaust background would be related to higher PTSD symptoms and intrusive linkage between the Holocaust and the Israel-Hamas War, (b) there will be reciprocal effects between PTSD symptoms, intrusive linkage, and sleep problems. That is, PTSD symptoms and intrusive linkage would predict more sleep problems (and sleep problems would predict more PTSD symptoms and intrusive linkage), (c) Holocaust background will have an indirect effect on sleep problems via higher PTSD symptoms and intrusive linkage.

## 2 Methods

### 2.1 Participants and procedure

A random sample of participants was recruited from across Israel by a web-based survey company (*iPanel, Israel*) in four waves. Inclusion criteria included being Israeli, Jewish, of European origin, and Hebrew-speaking adults born after 1945 (i.e., after World War II, WWII). Participants signed electronic informed consent before completing the questionnaire. Ethical approval was received from the Institutional Review Board of Ariel University (no. AU-SOC-LG-20230427 for T1, no. AU-SOC-LG-20231119 for T2, AU-SOC-LG-20240701 for T3, and no. AU-SOC-LG-20240628 for T4).

Following previous studies (e.g (28)), participants were divided into Holocaust descendants with at least one parent or grandparent who lived in countries dominated by the Nazi or pro-Nazi regime during WWII (1939-1945) and matched comparisons whose parents or grandparents did not reside in countries dominated by the Nazi or pro-Nazi regime during WWII. The participants in the present study were not related to one another, in contrast to previous studies in which familial relationships were confirmed through data collected directly by research assistants (e.g (28).,). In the current study, data were collected through a survey company; therefore, family relationships were not verified.

After the researchers’ rigorous examination of respondents’ familial backgrounds, 1, 071 participants participated in wave 1 (T1) during May-November 2022 (see (29, 30)), 706 in wave 2 (T2) during July 2023, 582 in wave 3 (T3) during December 2023 (two months after the October 7 attack), and 405 in wave 4 (T4) during July 2024.

The current study included 359 participants who completed waves T1-T4. The average age at T1 of the sample was 45.44 (*SD* = 12.85), 49.0% (*n* = 176) were women, and 60.4% (*n* = 217) had an academic degree. Two hundred twenty-four were descendants of Holocaust survivors, and 135 were comparison descendants. No significant differences in age, gender, or education level were found between the groups (*ps* ≥.096).

Attrition analysis showed that compared to participants who participated in T2 and T3 only (*n* = 131), those who completed all waves (*n* = 359) reported more sleep problems in T3 (*M* = 7.04, *SD* = 6.19 vs. *M* = 8.45, *SD* = 6.59, *t*(488]=-2.13, *p* = .03). However, the groups did not differ in all other variables including background characteristics, T2 sleep problems, T3 PTSD symptoms, or T3 intrusive linkage (*ps* ≥.19).

### 2.2 Measures

#### 2.2.1 T1 measures

2.2.1.1 Background characteristics assessed at T1 included age, gender, and education level, rated on a scale from 1 (*no formal education*) to 6 (*academic degree*).

2.2.1.2 Familial Holocaust background was determined in T1, relating to Holocaust survivors’ experiences during WWII. Participants provided information on their parents’ dates and places of birth (for the children) and their grandparents’ dates and places of birth (for the grandchildren). Items included the number of Holocaust survivors in the family, inhabited countries controlled by Nazi or pro-Nazi regimes during WWII, and experiences during the Holocaust (e.g., concentration camps, work camps, ghettos, hiding, using false papers, and escaping from authorities). The researchers conducted a thorough review of the data to accurately assign respondents to the appropriate study groups. Participants were subsequently categorized into Holocaust descendants (with a Holocaust survivor grand/parent) and comparison descendants (without a Holocaust survivor grand/parent).

#### 2.2.2 T2 measures

2.2.2.1 Sleep problems in T2 were assessed using the Pittsburgh Sleep Quality Index (PSQI (31),). Participants reported the quality of their sleep over the last month using 19 items (rated on various scales) that contributed to seven component scores, which were then summed to yield a global score. Higher scores indicated more sleep problems. Cronbach’s α was 0.76.

#### 2.2.3 T3-T4 measures

2.2.3.1 Traumatic exposure to the Israel-Hamas War was evaluated in T4 by six questions examining whether participants were: 1. evacuated from their home, 2. attacked by terrorists, 3. under missile attack, 4. in a situation in which they saw bodies, 5. personally familiar with injured people in the war, and 6. personally familiar with people who were killed in the war. The sum of the yes/no items was calculated.

2.2.3.2 PTSD symptoms in T3 and T4 were assessed by the International Trauma Questionnaire (ITQ (32);). The International Trauma Questionnaire (ITQ) is based on a clinical approach of the ICD-11 framework (33), with six items referring to the three symptom clusters of re-experiencing, avoidance, and sense of threat. Participants rated the symptoms on a scale ranging from 0 (*not at all*) to 4 (*very much*) while referring to the October 7 attack and the Israel-Hamas War. We calculated the sum of the items, with higher scores reflecting greater distress. Cronbach’s α was 0.83 and 0.87 at T3 and T4, respectively. Participants also reported whether the symptoms caused functional impairment on three additional items of important areas of life (32). These three items were also rated on a scale ranging from 0 (not *at all*) to 4 (“very much”). Following, probable PTSD was computed for descriptive purposes only. Following the algorithm for ICD-11 PTSD probable diagnosis (32), respondents who indicated a high level of suffering (≥2 on a 5-point scale) in at least one item of each of the three clusters of re-experiencing, avoidance, and sense of threat, and in at least one item of the functional items, were designated as having probable PTSD.

2.2.3.3 Intrusive linkage between the Holocaust and the Israel-Hamas War (henceforth referred to as “intrusive linkage”) *in T3 and T4* was examined with six items adapted from the Intrusive Memory Questionnaire (34). Respondents were asked to rate to what degree they reflect on the linkage between the Holocaust, the October 7 attack, and the Israel-Hamas War, on a five-point Likert scale ranging from 1 (*completely disagree*) to 5 (*completely agree*). Items included, for example: “I often think about the linkage between the Holocaust and the current war” (see (2) for more details). The average of ratings was computed, with higher scores indicating more intrusive linkage. Cronbach’s α was 0.90 and 0.91 at T3 and T4, respectively.

2.2.3.4 Sleep problems in T3 and T4 were assessed with the Jenkins Sleep Scale (JSS (35),. Respondents rated how many days they had experienced sleep problems over the last month using 4 items on a 6-point Likert scale ranging from 0 (*not at all*) to 5 (*22–30 days*). We calculated the sum of the items, with higher scores reflecting more sleep problems. Cronbach’s α was 0.91 in both T3 and T4. The clinical level of sleep problems (cut-off score>11) was computed for descriptive purposes only.

### 2.3 Data analysis

**Figure 1:**
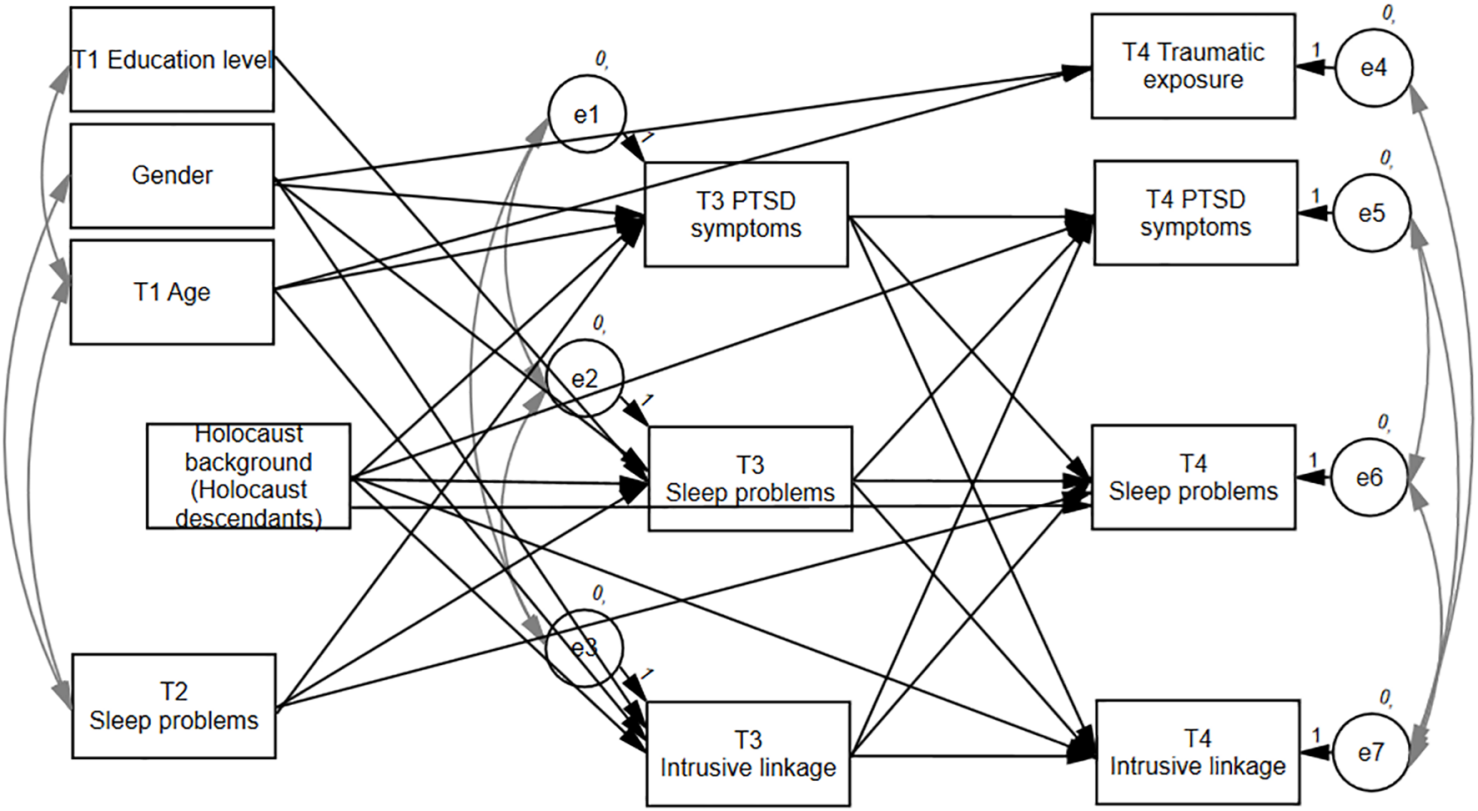
The study model.

Descriptive statistics and correlations were conducted using IBM SPSS Statistics (Version 31). We further conducted path analysis using AMOS 31 to examine the study hypotheses and the complex system of associations of PTSD symptoms, intrusive linkage, and sleep problems at T3 and T4. Path analysis provides a graphical representation of relationships among variables and can minimize Type 1 errors compared with analyses that focus only on single sets of associations. It also allows for the examination of covariates and reciprocal cross-lagged effects. In this analysis, T3 PTSD symptoms, intrusive linkage, and sleep problems were the predicting variables. Outcome variables were T4 PTSD symptoms, intrusive linkage, and sleep problems. We examined cross-lagged associations between T3 and T4 variables. We controlled for age, gender, education, traumatic exposure to the war, and T2 sleep problems. Correlations and covariate effects were retained only if significant. In cases where modification indices indicated that the goodness of fit improved by relating covariates to T4 variables, we allowed it. In addition, the model included the effect of Holocaust background (comparison descendants vs. Holocaust descendants) on T3/T4 PTSD symptoms, intrusive linkage, and sleep problems (see **Figure 1**). We employed the bootstrap technique with Gaskin and Lim’s AMOS plugin (36) to assess the significance of indirect effects, with Holocaust background as the independent variable, T3 variables as potential mediators, and T4 variables as outcomes.

Several indices were employed to determine whether the hypothesized models fit the data. A good model fit is indicated by (a) a non-significant chi-square, (b) comparative fit index (CFI) >.95, (c) root-mean-square error of approximation (RMSEA) <.06 (37).

## 3 Results

Descriptive statistics are presented in **Table 1**. Distress levels, reflected by PTSD symptoms and sleep problems, decreased from T3 to T4, as the war persisted after its initial traumatic surge. The prevalence of probable PTSD was 18.1% in T3 and 12.3% in T4 (McNemar *χ*^2^[1]=5.79, *p* = .01). The prevalence of clinical levels of sleep problems was 33.7% in T3 and 15.9% in T4 (McNemar *χ*^2^[1]=43.14, *p* <.001). Similarly, intrusive linkage declined as well (*t[*358]=9.13, *p* <.001).

**Table 1:** Descriptive statistics and correlations for the study variables.

Variable	*M/N*	*SD/%*	Range	1	2	3	4	5	6	7
1. PTSD symptoms T3	7.54	4.88	0-24	–						
2. Intrusive linkage T3	2.80	0.98	1-5	.53***	–					
3. Sleep problems T3	8.45	6.59	0-20	.63***	.39***	–				
4. PTSD symptoms T4	6.10	5.07	0-20	.64***	.37***	.52***	–			
5. Intrusive linkage T4	2.38	0.97	1-5	.48***	.59***	.43***	.54***	–		
6. Sleep problems T4	6.15	4.58	0-16	.53***	.37***	.70***	.57***	.49***	–	
7. Sleep problems T2	5.18	3.59	0-19	.27***	.11*	.46***	.31***	.21***	.49***	–
8. Holocaust background (Holocaust descendants) T1	224	62.4	0-1	.12*	.15**	.03	.02	.08	.07	.02
9. Age T1	45.44	12.85	19-68	-.21***	-.18***	.04	-.12*	-.09	.006	.20***
10. Gender (Woman) T1	196	49.0	0-1	.31***	.25***	.36***	.28***	.32***	.30***	.14**
11. Education (academic degree) T1	217	60.4	1-6	.06	.009	.20***	.11*	.08	.18***	.06
12. Traumatic exposure to the Israel-Hamas War T4	1.30	1.15	0-6	.20***	.05	.11*	.17***	.14*	.06	.09

*N* = 359. Correlations between categorical variable and a continuous variable were assessed with point-biserial correlations.

*p < .05, **p < .01, ***p < .001 (2-tailed).

The correlations showed that a Holocaust background was associated with higher T3 PTSD symptoms and intrusive linkage (meaning higher levels among Holocaust descendants). PTSD symptoms, intrusive linkage, and sleep problems showed strong within- and between-wave positive correlations. Moreover, some covariates showed moderate to strong correlations with the main variables. T2 sleep problems and gender (women) positively correlated with all the main variables in T3 and T4. Older age correlated with fewer PTSD symptoms in T3 and T4, but with more sleep problems in T2.

The parameters of the main model are presented in **Table 2**. The model showed good model fit, despite a significant chi-square, *χ*^2^(30) = 65.51, *p* <.001, CFI = .97, RMSEA = .05 (90%CI:.03,.07). Holocaust background predicted higher PTSD symptoms and intrusive linkage in T3, meaning that these variables were higher among Holocaust descendants. T3 PTSD symptoms predicted more intrusive linkage and sleep problems in T4. T3 intrusive linkage predicted more sleep problems in T4. Moreover, T3 sleep problems predicted more PTSD symptoms and intrusive linkage in T4.

**Table 2:** Overview of parameters of the study model.

Regression weights			B	*p*	β	SE
Holocaust background						
Holocaust background (Holocaust)	→	PTSD symptoms T3	**0.91**	**.049**	**.09**	**.46**
Holocaust background (Holocaust)	→	Intrusive linkage T3	**0.27**	**.006**	**.13**	**.10**
Holocaust background (Holocaust)	→	Sleep problems T3	0.36	.53	.02	.59
Holocaust background (Holocaust)	→	PTSD symptoms T4	-0.48	.24	-.04	.41
Holocaust background (Holocaust)	→	Intrusive linkage T4	-0.03	.66	-.01	.08
Holocaust background (Holocaust)	→	Sleep problems T4	0.18	.57	.02	.33
Sleep problems T2						
Sleep problems T2	→	PTSD symptoms T3	**0.31**	**<.001**	**.23**	**.05**
Sleep problems T2	→	Sleep problems T3	**0.70**	**<.001**	**.39**	**.07**
Sleep problems T2	→	Sleep problems T4	**0.24**	**<.001**	**.19**	**.04**
Auto-regression parameters, T3-T4						
PTSD symptoms T3	→	PTSD symptoms T4	**0.54**	**<.001**	**.52**	**.05**
Intrusive linkage T3	→	Intrusive linkage T4	**0.46**	**<.001**	**.47**	**.04**
Sleep problems T3	→	Sleep problems T4	**0.35**	**<.001**	**.50**	**.03**
Cross-lagged parameters, T3-T4						
PTSD symptoms T3	→	Intrusive linkage T4	**0.02**	**.04**	**.11**	**.01**
PTSD symptoms T3	→	Sleep problems T4	**0.10**	**.02**	**.11**	**.04**
Intrusive linkage T3	→	PTSD symptoms T4	0.14	.54	.02	.23
Intrusive linkage T3	→	Sleep problems T4	**0.44**	**.02**	**.09**	**.19**
Sleep problems T3	→	PTSD symptoms T4	**0.14**	**<.001**	**.18**	**.03**
Sleep problems T3	→	Intrusive linkage T4	**0.02**	**<.001**	**.17**	**.008**

*N* = 359. The model also included age, gender, education, and traumatic exposure to the war. The estimates for covariates and correlations are not presented to maintain the table's succinctness. Values in bold represent significant results.

Several significant indirect effects were found. First, there was an indirect effect of Holocaust background via T3 PTSD symptoms on T4 intrusive linkage (estimate=0.02, 95%CI: 0.00, 0.06, *p* = .04). Meaning, Holocaust descendants reported higher PTSD symptoms in T3, which in turn predicted more intrusive linkage in T4.

Second, there was an indirect effect of Holocaust background via T3 PTSD symptoms on T4 sleep problems (estimate=0.09, 95%CI: 0.00, 0.30, *p* = .04). Finally, there was an indirect effect of Holocaust background via T3 intrusive linkage on T4 sleep problems (estimate=0.12, 95%CI: 0.01, 0.31, p=.02). Meaning, Holocaust descendants reported higher PTSD symptoms and intrusive linkage in T3, which in turn predicted more sleep problems in T4.

## 4 Discussion

Our initial findings that intrusive linkage and especially PTSD symptoms and sleep problems decreased between T3 and T4 indicate a reduction in distress between the period close to the 2023 October 7 attack on Israel and about a year and a half later. In addition, results confirm that descendants of Holocaust survivors report more PTSD symptoms and more intrusive linkage than descendants of non-Holocaust survivors. As expected, reciprocal relations were found among PTSD symptoms, intrusive linkage, and sleep problems. Most importantly, PTSD symptoms mediated the relation between being descendants of Holocaust survivors and later intrusive linkage. PTSD and intrusive linkage mediated the relation between being descendants of Holocaust survivors and later sleep problems.

Findings support the first hypothesis that ancestral trauma can be linked to current PTSD symptoms and intrusive linkage even over intergenerational transmission. The results that a Holocaust background was associated with higher PTSD symptoms following the 2023 October 7 attack and Israel-Hamas war, as well as higher intrusive linkage, are in line with previous results showing that a Holocaust background predicts secondary traumatization and event centrality (28). Descendants of Holocaust survivors have previously reported higher levels of salience to adverse events. Examples of this relationship include the Russo-Ukrainian war (30), the Iranian nuclear threat to Israel (27), and the anxiety over ISIS (8).

The results showing that intrusive linkage can bridge between ancestral trauma and personal trauma are important for two reasons: First, the results extend Dashorst et al.’s (34) original idea that one may have intrusive memories of their ancestral trauma. Moreover, this extends Shrira et al.’s (2) application of this concept from a one-person model to an intergenerational transmission between two people (parents and children). Herein, we show that one’s ancestral trauma predicts more intrusive linkage, which predicts higher PTSD symptoms following a current trauma experienced by the offspring. The second extension adds to the previously suggested mechanisms of interventional transmission (38, 39). For example, parental behavior was found to be an impactful mechanism linking parental trauma with transmission of trauma to offspring (40, 41). Another intergenerational transmission mechanism can be parental communication, which may intensify stress and diminish coping (42). When communication emphasizes a catastrophizing absence of delineation between past and present calmatives, intergenerational transmission may increase (43). Other mechanisms that may play a role in determining whether parental trauma will manifest in future generations include the centrality of Holocaust trauma into offspring identity (44), attachment (45), and epigenetic mechanisms (46).

Distress levels, indicated by PTSD symptoms and sleep problems, decreased from the higher levels measured two-four months after the 2023 October 7 attack to lower levels reported about a year and a half later. Intrusive linkage also declined during this period. Additionally, as expected, we found strong within- and between-wave (positive) correlations of PTSD symptoms, intrusive linkage, and sleep problems. Importantly, sleep problems measured three months before the attack predicted PTSD symptoms and intrusive linkage in T3 and T4. PTSD symptoms and intrusive linkage at T3 predicted sleep problems a year and a half later. Therefore, our findings confirm the reciprocal nature of the relations among PTSD symptoms, intrusive linkage, and sleep problems.

However, the current results showing that ancestral trauma predicted higher PTSD symptoms of the October 7 attack in offspring, which in turn predicted higher intrusive linkage, seem to present a novel mechanism of intergenerational transmission, which was not previously addressed. This interpretation also benefits from the finding that intrusive linkage mediates the effects of ancestral trauma on sleep problems. Concerning our next hypothesis, the findings suggest an indirect effect of ancestral trauma on current sleep problems after exposure to a traumatic event, through PTSD symptoms and through intrusive linkage. That is, being descendants of Holocaust survivors predicted higher PTSD symptoms two months after the October 7 attack, and PTSD symptoms two months after the October 7 attack predicted sleep problems a year and a half later. Placing intrusive linkage reports as a mediator between Holocaust ancestral trauma and subsequent sleep problems, a year and a half after the 2023 October 7 attack and into the Israel-Hamas war, yielded the same pattern of results.

Two mechanisms mentioned above were mentioned to explain how PTSD symptoms can drive sleep problems. First, the PTSD–insomnia association builds on the hyperarousal symptoms of PTSD (18). Second, Lemyre et al. (20) emphasize the role of intrusive thoughts in insomnia. In this regard, intrusive thoughts due to PTSD elevate the risk for insomnia. This result of intrusive linkage mediating between Holocaust background and subsequent sleep problems may add support to the latter explanation. It may also highlight the important role of intrusive thoughts in triggering distress. Future research is needed to examine the properties and limits of such a mechanism, which should help reveal conditions under which intergenerational and other traumas are transmitted.

The associations in the direction of sleep problems to PTSD symptoms and sleep problems to intrusive linkage can be similarly explained. Sleep loss can influence executive function capabilities (47), consequently reducing the ability to inhibit maladaptive intrusive thoughts, thereby elevating the risk of PTSD (22). The intrusive linkage can probably be linked to the same rationale, as it relates to thoughts that emerge and preoccupy a person when internal control is diminished due to impaired executive functions arising from sleep problems. Additionally, insufficient sleep may elevate the risk of PTSD symptoms because of inadequate emotional memory consolidation, which in turn may render the trauma memories insufficiently processed, thereby contributing to the development of PTSD (23, 26, 48).

Some caveats should be considered concerning this study. We do not have first-hand information about the nature of the experiences the first-generation person had, if and how many, or for how long, that person experienced traumatic events. Thus, the findings are considered probable trauma. However, the results in which Holocaust descendants show more trauma vulnerability are robust and merit an explanation beyond the mere fact that the participants are descendants of Jewish people who lived in Europe. Our knowledge of the horrors of that period justifies an interpretation of probable trauma as actually describing the reality of the lives of that generation. Additionally, as Israelis, comparison participants were educated and knowledgeable about the Holocaust period. Since differences in emotional responses have been documented, personal linkage, rather than mere knowledge, seems to be a reasonable consideration. Second, the study is based on a panel sample in which participants shared a common background. This probably led to narrowing socio-demographic characteristics. In addition, assessing intrusive linkage together with psychopathology after the attack may lead to some bias in the reports. Future research can try to discriminate it from psychopathology by assessing more time-lagged effects.

## Data Availability

The raw data supporting the conclusions of this article will be made available by the authors, without undue reservation.
